# Long non-coding RNA HOTAIR functions as a competitive endogenous RNA to regulate PRAF2 expression by sponging miR-326 in cutaneous squamous cell carcinoma

**DOI:** 10.1186/s12935-019-0992-x

**Published:** 2019-10-21

**Authors:** Guo-Jun Yu, Yong Sun, Da-Wei Zhang, Peng Zhang

**Affiliations:** 1 Department of Burn and Plastic Surgery, The Affiliated Huaian No.1 People’s Hospital of Nanjing Medical University, 6 Beijing Xi Road, Huaian, 223300 Jiangsu China; 2 Department of ICU, The Affiliated Huaian No.1 People’s Hospital of Nanjing Medical University, Huaian, Jiangsu China

**Keywords:** Cutaneous squamous cell carcinoma, lncRNA, ceRNA, Cell migration

## Abstract

**Background:**

LncRNAs may exert a regulatory effect in tumorigenesis. Although the expression of lncRNA *HOTAIR* has been confirmed to be notably elevated in the tissues of CSCC, its biological mechanism in CSCC is still unknown.

**Methods:**

*HOTAIR* expression level in CSCC cell lines was monitored via qRT-PCR. Then CCK-8 assay, Transwell assay and EdU assay were adopted to detect cell migration and proliferation. Meanwhile, through bioinformatics analysis and luciferase reporter gene detection, a new target of *HOTAIR* was identified. Additionally, Western blotting and RIP analysis were adopted to discuss the possible mechanism.

**Results:**

*HOTAIR* expression in CSCC cell lines exhibited an obvious elevation. Cell function analysis revealed that *HOTAIR* overexpression remarkably facilitated CSCC cell migration, proliferation and EMT process, which were impeded by down-regulation of *HOTAIR*. Furthermore, *HOTAIR* competitively bound to miR-326, so as to positively modulate miR-326 expression.

**Conclusions:**

These results present that *HOTAIR*, as a ceRNA, regulates *PRAF2* expression by competitive binding to miR-326 during CSCC.

## Background

The dermis and epidermis constitute the skin, the largest organ [1]. As the outer layer of the skin, the epidermis directly contacts with external particles, pathogens, UV rays and chemicals in the environment [2]. DNAs will mutate due to exposure of skin cells to UV rays and chemicals for a long time, which may induce the development of cancers, so skin cancer becomes the most common cancer in human, especially in those with low level of melanin in the skin [3, 4]. Non-melanoma skin cancer (NMSC) and melanoma are two common types of skin cancers. NMSC can be classified into basal cell carcinomas (BCCs) and cutaneous squamous cell carcinoma (CSCC) [5]. According to the estimation of the American Academy of Dermatology, there are about 9500 new cases of skin cancer every day and more than 3 million Americans annually suffered from NMSC [6]. CSCC, a skin tumor derived from epidermal keratinocytes, ranks second among frequently occurring NMSC types. Emphasis in current research on CSCC has been gradually put on the gene, molecule and protein level. For instance, Mei [7] discovered that long non-coding RNA (lncRNA) *LINC00520* impedes CSCC to progress through inactivating the PI3K/Akt signaling pathway by down-regulation of *EGFR*. Gong et al. [8] showed that miR-221 accelerates the progression of CSCC through targeting *PTEN*. In order to improve people’s understanding of CSCC, the development and metastasis mechanisms of CSCC were further explored from the perspective of epigenetics, which will be conducive to improving the existing diagnosis and treatment methods and have important clinical significance.

LncRNAs refer to ncRNAs that can regulate gene expression with 200 nt in length [9, 10]. In recent years, lncRNAs have aroused extensive attention of researchers due to its complex biological functions. Besides, they have been confirmed to exert pivotal effects on proliferation, apoptosis, invasion and infiltration of many malignant tumor cells [11–14]. As a lncRNA, *HOTAIR* (Gene ID: 100124700) is located in the 12q13.13 region of the human genome, which is crucial in the pathological process of a variety of diseases, such as endocrine system diseases [15], cardiovascular diseases [16] and diverse tumors [17]. *HOTAIR* has been verified to be involved in the occurrence mechanisms of cervical cancer [18] and breast cancer [19] by promoting tumor cell migration and proliferation. Through expression profiles and qRT-PCR assay, Sand et al. [20] proved that *HOTAIR* is up-regulated in CSCC tissues compared with nonlesional epithelial skin. Although it has been found that lncRNA *HOTAIR* is related to the pathological process of CSCC, the exact mechanism of *HOTAIR* in participating in the occurrence process of CSCC still needs to be explored.

Recently, emerging evidence indicated the crucial roles of miRNAs in various human diseases [21–25]. Muhammad et al. [26], found that Anti-miR-203 suppresses ER-positive breast cancer growth and stemness by targeting SOCS3. Gong [8] stated that miRNA-221 promotes cutaneous squamous cell carcinoma progression by targeting PTEN. miR-326 functions as a tumor suppressor in gastric cancer [27], lung cancer [28], breast cancer [29] and so on. Nevertheless, whether miR-326 can inhibit the progression of CSCC needs further study.

Our research team verified that *HOTAIR* exhibited a high expression in CSCC cell lines, and elevated *HOTAIR* stimulates the migration and proliferation of A431 and SCL-1. Overall, results presented that *HOTAIR* competitively bound to miR-326, so as to affect the expression of prenylated Rab acceptor 1 domain family, member 2 (*PRAF2*) and participate in the mechanism of CSCC, thus creating an option for studying CSCC based on lncRNAs.

## Methods

### Culture and transfection of cells

HaCaT (one human keratinocyte cell line) and A431, HSC-5, SCC13, and SCL‐1 (four CSCC cell lines) were bought from Shanghai Cell Bank, Chinese Academy of Sciences (Shanghai, China). Then the cells were cultured in DMEM (Gibco, Grand Island, NY) containing 10% FBS (Beyotime, Nantong, China) as well as 100 μg/mL streptomycin and 100 IU/mL penicillin (Invitrogen, USA) in a cell incubator with 5% CO_2_ at 37 °C. *HOTAIR* overexpression (*HOTAIR* OE) plasmids, *HOTAIR* siRNAs, miR-326 mimics and miR-326 inhibitors were synthesized by GenePharma (Shanghai, China). On the basis of manufacturer’s protocol, Lipofectamine 2000 (Invitrogen, CA, USA) was applied to transfect cells.

### RNA extraction and qRT-PCR

For total RNA extraction from cells, TRIzol reagent (Takara, Tokyo, Japan) was used following the manufacturer’s protocol. For cell lysis, the cells were washed with PBS and 1 mL TRIzol was added per well for 3 min. The concentration and purity of the RNA were evaluated using a spectrophotometer (Bio-Rad, Hercules, CA). The Reverse Transcription Kit (Takara, Tokyo, Japan) was utilized for transcription of RNA to cDNA. Afterwards, real-time quantitative PCR (qRT-PCR) was performed using the SYBR Green PCR Master Mix (Invitrogen, USA). RNA was quantified through normalizing to GAPDH using 2^−ΔΔCt^ method. PCR primers used are displayed in Table 1. Each experiment was independently conducted for three times.

**Table 1:** Sequences of primers for qRT-PCR

Name	Sequence
lncRNA-HOTAIR	
Forward	5′-GAGGGGAGCAGAGTTCAAGT-3′
Reverse	5′-TGGGAGGCAGCAATAGACAA-3′
PRAF2	
Forward	5′-CTGGACGACTTTGTTCTGGGG-3′
Reverse	5′-GCTCAGGAGCGTATGAAGTGG-3′
GAPDH	
Forward	5′-GCACCGTCAAGGCTGAGAAC-3′
Reverse	5′-GGATCTCGCTCCTGGAAGATG-3′
U6	
Forward	5′-CTCGCTTCGGCAGCACA-3′
Reverse	5′-AACGCTTCACGAATTTGCGT-3′
miR-326	
Forward	5′-ACACTCCAGCTGGGGACCTCCTTCCCGG-3′
Reverse	5′-CTCAACTGGTGTCGTGGAGTCGGCAATTCAGCCCAGAGG -3′

### Cell proliferation assay

To detect cell proliferation, CCK-8 (Beyotime, Nantong, China) assay was conducted in accordance with manufacturer’s regimen. On 96-well plates, the cells were subjected to 24 h of culture, addition of CCK-8 and 1 h of incubation in a cell incubator. Cell optical density at 450 nm was identified by means of the Tecan infinite M200 multimode microplate reader (Tecan, Mechelen, Belgium). Next, the proliferation of cells was monitored via EDU assay. Then the cells were transfected, incubated in EdU medium for 2 h, and fixed in 4% paraformaldehyde for 15 min. EdU staining was carried out with reference to manufacturer’s instructions.

### Cell migration examination

Cell migration capacity was examined via Transwell assay. At 24 h after transfection, 1 × 10^6^ cells suspended in 100 μL medium free of serum were added to the top chamber, and 600 μL medium with 10% FBS to the bottom chamber. Then the cells were incubated for 24 h, fixed in 4% polymethyl alcohol for 15 min, dyed with 0.1% crystal violet (Beyotime, Nantong, China) for 20 min, and photographed with a microscope (×200, ten views per well). Additionally, the transferred cells were quantified using Image-Pro Plus 6.0 (Media Cybernetics, USA).

### TUNEL staining

In accordance with the manufacturer’s instructions, TUNEL assays were performed on xenograft tumor tissues using an Apoptosis Detection Kit (Ribobio, China). TUNEL-positive cells were evaluated in a randomly selected field of view with no significant necrosis. The TUNEL index was calculated based on the total number of nuclei and cells with green nucleus. All samples were assayed in triplicate.

### Wound healing assay

Scratch wound healing was applied to measure cell migration. On 6-well plates, the cells were inoculated and scratched using a pipette tip (10 μL). After washing thrice, the non-adherent cells were washed. Cell migration toward the wound 24 h post-scratching was photographed by using an inverted microscope (Olympus, Japan), and the total wound areas were analyzed on Image J to assess migration capacity.

### Cell apoptosis and cell cycle assays

Annexin V-FITC and propidium iodide (PI) were applied to label cells in cell apoptosis assay, and then the cells were subjected to flow cytometry (BD Biosciences, Franklin Lakes, NJ, USA). Flow cytometry was adopted to examine the cells stained by PI in cell cycle assay. In both assays, a flow cytometer (FACScan; BD Biosciences, USA) equipped with Cell Quest software (BD Biosciences) was applied.

### Subcellular fractionation location

RNAs in the nucleus and cytoplasm were separated with the PARIS Kit (Life Technologies, USA) based on the manufacturer’s instructions. As mentioned above, the total RNAs extracted from each fraction were determined via qRT-RCR. GAPDH was taken as a cytoplasmic marker, while U6 as a nuclear control transcript.

### Dual-luciferase reporter gene assay

Wild-type plasmid *HOTAIR*-WT, *PRAF2*-WT and mutant plasmid *HOTAIR*-MUT, *PRAF2*-MUT were constructed. A431 and SCL-1 on 24-well plates were co-transfected with 50 nM miR-326 mimics or NC and wild-type or mutant plasmid using Lipofectamine 2000. Then pRL-SV40 was added into plasmids at the ratio of 1:16. Dual-luciferase reporter assay kit (Promega, Madison, WI, USA) was used for determining luciferase intensity on a microplate reader.

### RNA-binding protein immunoprecipitation (RIP) analysis

In RIP assay, the Magna Nuclear RIP™ (Native) RIP Kit (Millipore, Bedford, MA, USA) was utilized. Cells underwent lysis in complete RIPA buffer with an RNase inhibitor and protease inhibitor cocktail. Then magnetic beads in RIP buffer were conjugated to immunoglobulin G (IgG) control or human anti-AGO2 antibody (Millipore), and the buffer was used to incubate the cell extract. Immunoprecipitated RNA was obtained from protein digestion. Finally, the purified RNA was quantified by qRT-PCR. Anti-*HOTAIR* applied for RIP assay was bought from Abcam (Cambridge, MA, USA).

### Western blotting analysis

RIPA was used to extract total proteins. SDS-PAGE gel with appropriate concentration was selected in light of the molecular weight of target proteins. After electrophoresis, the proteins were transferred onto PVDF membranes, and primary antibodies (Abcam, Cambridge, USA) of *PRAF2*, *E*-*cadherin*, *N*-*cadherin*, *Twist*, *Snail1*, *ZEB*-*1*, *Cyclin D1*, *pro*-*caspase*-*3*, *cleaved*-*caspase*-*3* and *β*-*actin* were applied to incubate these membranes. The anti-rabbit or anti-mouse HRP-linked secondary antibodies (diluted at 1:1000; Beyotime, Nantong, China) were added for 2 h of incubation at 37 °C. Data analysis was carried out with ImageJ software (NIH, Washington, DC, USA).

### Immunohistochemistry

Immunohistochemical staining was performed according to published methods [30]. First, 3 μm paraffin sections of tissue samples were stained with immunohistochemistry. The primary antibody specific for Ki-67 (Abcam, Cambridge, USA) was used at a 1:100 dilution in the experiments. Images were captured using a Nikon Eclipse 80i system with NIS-Elements software (Nikon, Japan).

### Animal experiments

The flanks of BALB/c thymic free nude mice (female, 4–6 weeks old, Animal Center of Shanghai Jiaotong University) were subcutaneously injected with A431. After cell injection for 4 weeks, the mice were executed and tumors were excised for analysis. This study was approved by the ethic committee in The Affiliated Huaian No.1 People’s Hospital of Nanjing Medical University, and experiments were performed according to the animal welfare and NIH requirement.

### Statistical analysis

Statistical analysis was conducted by means of SPSS20.0 software (SPSS, Chicago, IL, USA) and GraphPad Prism 6.0 (GraphPad Software Inc., CA, USA). Student’s *t*-test was performed to assess the statistical difference among data sets when the data conformed to the normal distribution, while the nonparametric test was used when the data did not accord with the normal distribution in all relevant experiments. The multigroup comparisons were determined by one-way ANOVA followed by Dunnett’s multiple comparison test. All data were presented as mean ± SD. The difference would be statistically significant when *P*< 0.05.

## Results

### Role of *HOTAIR* in CSCC cell lines

**Fig. 1:**
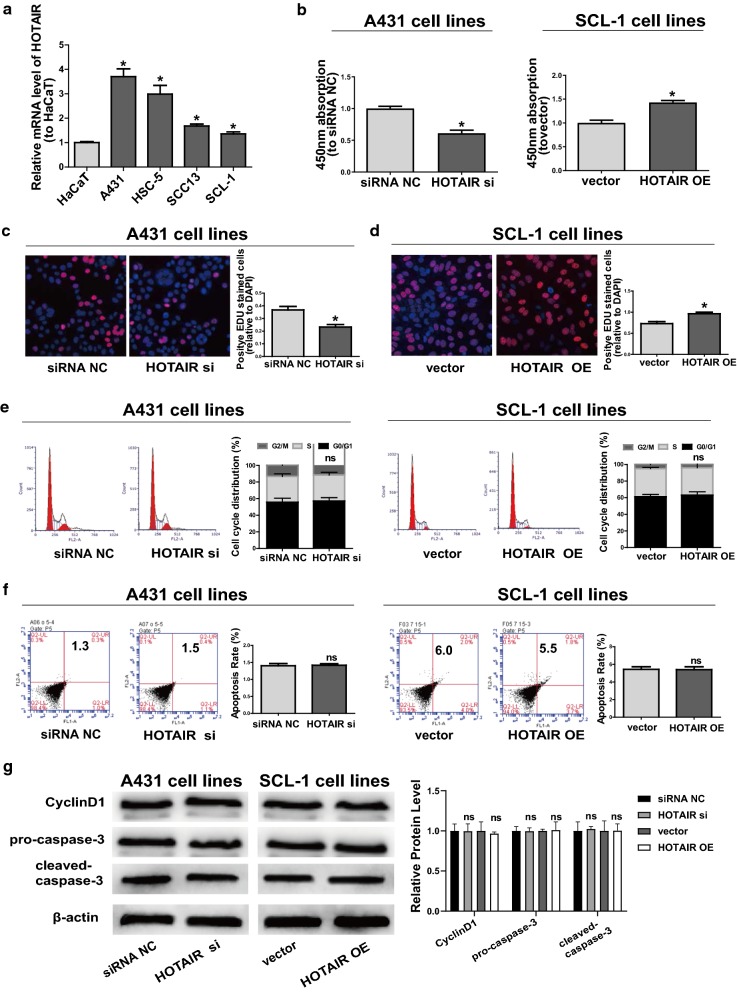
Regulatory effects of *HOTAIR* on proliferation, apoptosis and cell cycle progression of CSCC cells. **a** Detection of *HOTAIR* expressions in CSCC cell lines (A431, SCL-1, HSC-5, and SCC13) and one human keratinocyte cell line (HaCaT) via qRT-PCR. **b** CCK-8 assay shows the proliferation of A431 transfected with *HOTAIR* siRNAs and SCL-1 transfected with *HOTAIR* OE plasmids. **c**, **d** EdU assay shows the proliferation of A431 transfected with *HOTAIR* siRNAs and SCL-1 transfected with *HOTAIR* OE plasmids. **e** Determination of cell cycle through BD Biosciences FACSCalibur™ Flow Cytometry. **f** Detection of cell apoptosis through BD Biosciences FACSCalibur™ Flow Cytometry. **g** Detection of the expression of *Cyclin D1*, *pro*-*caspase*-*3*, *cleaved*-*caspase*-*3* through western blot. Data are presented as mean ± SD. *P < 0.05; ns, no significant difference; siRNA NC, siRNA negative control group; vector, overexpression plasmid vector negative control group

**Fig. 2:**
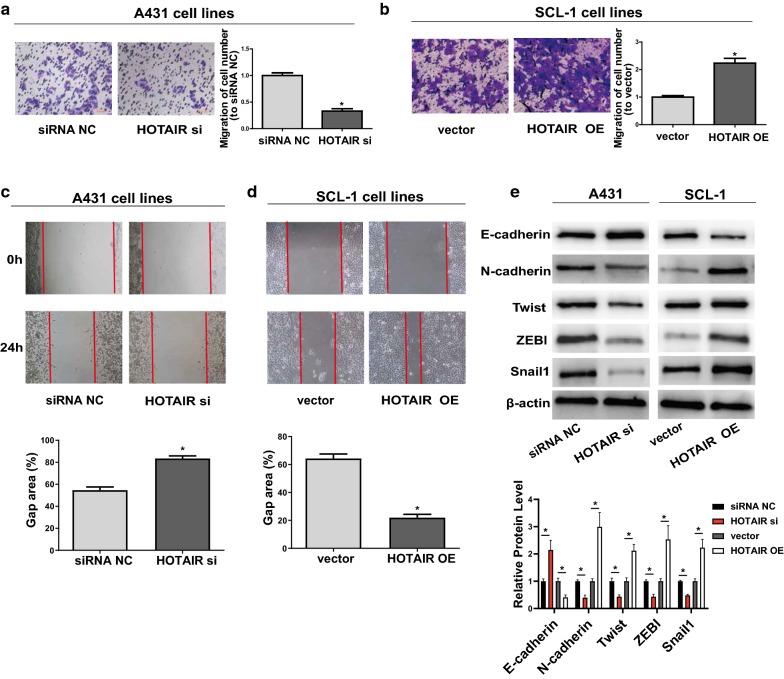
Regulatory effects of *HOTAIR* on cell migration and progression of CSCC cells. **a**, **b** Transwell assay shows the migration of A431 transfected with *HOTAIR* siRNAs and SCL-1 transfected with *HOTAIR* OE plasmids. Images are harvested under a light microscope (×200). **c**, **d** Wound healing assay shows the migration of A431 transfected with *HOTAIR* siRNAs and SCL-1 transfected with *HOTAIR* OE plasmids. **e** Transfection with *HOTAIR* siRNAs in A431 promotes the expression of *E*-*cadherin* and inhibited the expressions of *N*-*cadherin*, *Twist*, *Snail1 and ZEB1*. Transfection with *HOTAIR* OE plasmids in SCL-1 represses the expression of *E*-*cadherin* and promotes *N*-*cadherin* and *Twist* expressions. Data are presented as mean ± SD. *P < 0.05; siRNA NC, siRNA negative control group; vector, overexpression plasmid vector negative control group

To elucidate whether *HOTAIR* was elevated in CSCC cell lines, *HOTAIR* expression level in CSCC cell lines and a human keratinocyte cell line (HaCaT) was measured via qRT-PCR. It could be seen from Fig. 1a that compared with HaCaT, the level of *HOTAIR* in CSCC cells was increased remarkably, which is identical to the findings of Sand [20] in CSCC tissues. In the meantime, *HOTAIR* had the highest expression in A431 and the lowest expression in SCL-1 compared with that in other CSCC cell lines. Hence, A431 and SCL-1 were selected as research objects in the following experiments. Subsequently, qRT-PCR was conducted to verify transfection efficiency, which manifested that *HOTAIR* siRNAs were capable of prominently interfering with the expression of *HOTAIR* while *HOTAIR* OE plasmids increased its expression remarkably (Additional file 1: Figure S1A, B). Meanwhile, it was proved by CCK-8 assay that down-regulation of *HOTAIR* (*HOTAIR* siRNAs) evidently attenuated the proliferation ability of CSCC cells. In contrast, *HOTAIR* OE obviously enhanced proliferation ability of CSCC cells (Fig. 1b). Results of EdU assay were consistent with those of CCK-8 (Fig. 1c, d). However, neither down-regulation nor *HOTAIR* OE would affect the apoptosis and cycle of CSCC cells (Fig. 1e, f), so as the expression of *Cyclin D1*, *pro*-*caspase*-*3*, *cleaved*-*caspase*-*3* (Fig. 1g). Moreover, cell migration experiments showed that down-regulation of *HOTAIR* notably weakened the migration ability of CSCC cells (Fig. 2a), while *HOTAIR* OE prominently promoted its migration (Fig. 2b), which were also confirmed by wound healing assay (Fig. 2c, d). EMT process is known to be closely associated with cell migration. Subsequently, the expressions of mesenchymal marker *N*-*cadherin*, epithelial marker *E*-*cadherin* and EMT-related markers *Twist*, *Snail1 and ZEB1* were measured. It could be seen that *HOTAIR* siRNAs could elevate *E*-*cadherin* expression but inhibit the expressions of *N*-*cadherin* and *Twist*, *Snail1 and ZEB1*. Transfection with *HOTAIR* OE plasmids had an expected opposite effect (Fig. 2e). All in all, these results reveal that *HOTAIR* may exert regulatory effects on CSCC cell migration and proliferation to a certain degree.

### *HOTAIR* promotes CSCC cell proliferation in vivo

**Fig. 3:**
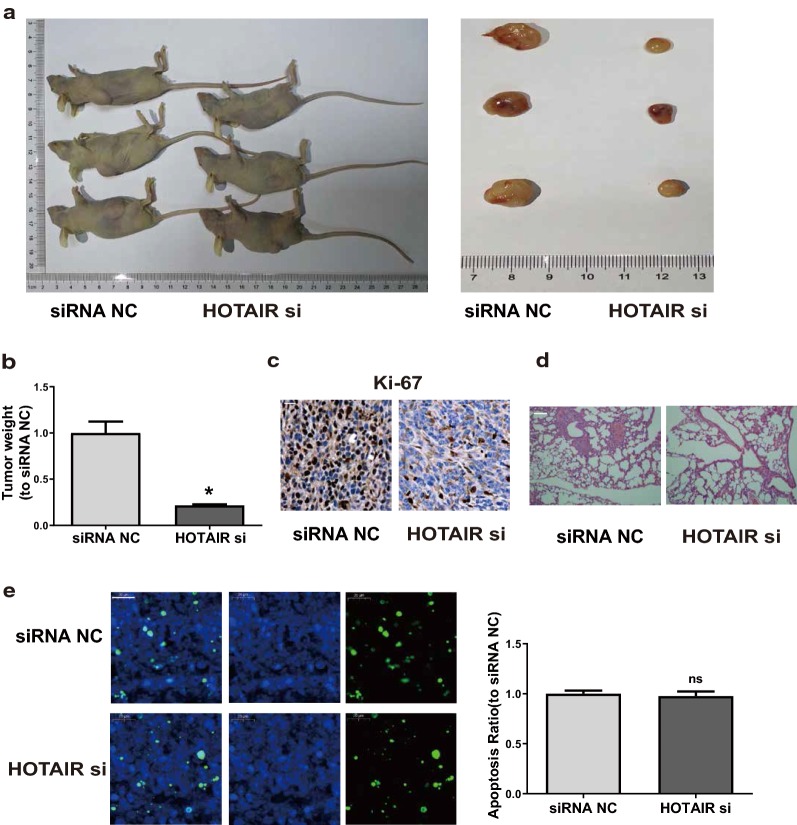
*HOTAIR* promotes CSCC cell proliferation in vivo. **a** Representative images of xenografts tumor in nude mice. **b** Tumor weight is monitored. **c** Representative images of IHC stained Ki-67 are shown (bar = 20 μm). **d** Representative images of H&E stained lung tissues are shown. **e** Representative images of TUNEL stained tumors tissues are shown (bar = 20 μm). Data were presented as mean ± SD. *P < 0.05; ns, no significant difference; siRNA NC, siRNA negative control group

To explore the role of *HOTAIR* in tumor growth of CSCC in vivo, nude mice received subcutaneous injection of A431 transfected with NC or *HOTAIR* siRNAs. The results displayed that down-regulation of *HOTAIR* decreased the tumor volume (Fig. 3a) and tumor weight (Fig. 3b) after the 4-week intratumorally injection. Furthermore, immunohistochemistry demonstrated that mice received subcutaneous injection of A431 transfected with si-*HOTAIR* appeared to have a lower level of Ki-67, the proliferation-specific gene in mice tumor tissues (Fig. 3c). Interestingly, after removing the lung tissues of nude mice, we found that the destruction of lung tissues was more pronounced in control group compared to the si-*HOTAIR* group (Fig. 3d). Then, we performed TUNEL assay to examine the apoptotic cells in the tumors tissues as shown in new Fig. 3e. Results showed that there is no significant difference between the control and HOTAIR siRNA group.

### Subcellular localization of *HOTAIR*

**Fig. 4:**
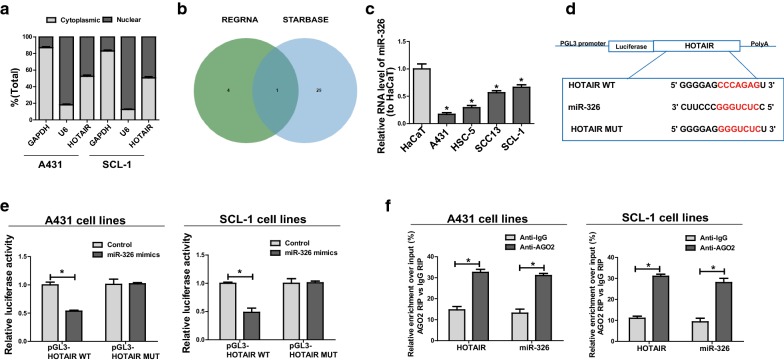
*HOTAIR* directly interacts with miR-326. **a** Cytoplasmic and nuclear levels of *HOTAIR* in A431 and SCL-1 analyzed by qRT-PCR. **b** Bioinformatics predictions from RegRNA and Starbase. **c** MiR-326 expression in CSCC cell lines and HaCaT detected by qRT-PCR. **d** Binding of miR-326 onto 3′-UTR of *HOTAIR* evidenced by bioinformatics. **e** Dual-luciferase reporter gene assay in A431 and SCL-1 after transfection with NC or miR-326 mimics, Renilla luciferase plasmid pRL-SV40 and the reporter constructs. **f** Detection of the amount of *HOTAIR* and miR-326 in A431 and SCL-1 via RIP experiments. Data are presented as mean ± SD. *P < 0.05

Subcellular localization of lncRNA is known to determine how lncRNA functions. In order to elucidate the cellular localization of *HOTAIR*, CSCC cells were isolated into the cytoplasm and the nucleus, with U6 and GAPDH as controls. U6 is mainly distributed in the nucleus, whereas GAPDH mainly exists in the cytoplasm. According to qRT-PCR results, 52.5% and 50.6% *HOTAIR* were detected in the cytoplasm of A431 and SCL-1, respectively (Fig. 4a), displaying that *HOTAIR* may be located in both cytoplasm and nuclei of CSCC cells. These results meant that *HOTAIR* may be involved in transcriptional and post-transcriptional level regulation. Considering that more than half of HOTAIR located in the cytoplasm of CSCC cells, we mainly explored its function in post-transcriptional level in the mechanism of CSCC.

### *HOTAIR* is targeted by miR-326

Although *HOTAIR* was discovered to be markedly up-regulated in CSCC cells and to speed up migration and proliferation CSCC cells, the exact mechanism of *HOTAIR* in participating in CSCC is still unclear. It was inferred that *HOTAIR* might act as a (competing endogenous RNA) ceRNA in biological processes [31, 32]. Through the intersection of RegRNA and Starbase software prediction results, it was found that miR-326 highly matched with the sequences of *HOTAIR* in 3′-UTR (Fig. 4b). QRT-PCR manifested that miR-326 expression level in CSCC cells was reduced (Fig. 4c), which was contrary to the expression trend of *HOTAIR* in CSCC cells. To study the interaction between *HOTAIR* and miR-326, *HOTAIR* fragments containing mutant or predicted target sites were established into the downstream of the firefly luciferase gene (pGL3-*HOTAIR*-WT and pGL3-*HOTAIR*-MUT) (Fig. 4d). The relative luciferase expression declined remarkably in SCL-1 and A431 co-transfected with *HOTAIR*-WT and miR-326 mimics, but luciferase intensity did not notably change after transfection with *HOTAIR*-MUT (Fig. 4e). Moreover, whether miRNA-containing ribonucleoprotein complexes involved *HOTAIR* was demonstrated via RIP assay in SCL-1 and A431. Then the relative RNA expression in immunoprecipitates was measured via qRT-PCR, which revealed that a large number of *HOTAIR* RNAs were enriched by anti-AGO2 antibodies in cells in contrast to IgG control. The expected similar results were obtained at miR-326 (Fig. 4f). The above-mentioned results suggest that HOTAIR directly interacts with miR-326.

### *HOTAIR* regulates miR-326 target gene, *PRAF2*

**Fig. 5:**
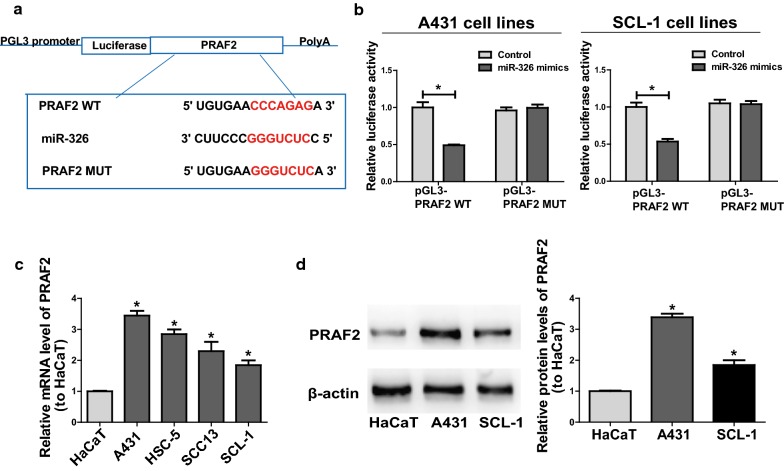
*PRAF2* is the direct target of miR-326. **a** The putative miRNA binding sites in the *PRAF2* sequence. **b** Dual-luciferase reporter gene assay is carried out to verify the direct target sites. **c** *PRAF2* expression in CSCC cell lines and HaCaT detected by qRT-PCR. **d** Protein levels of *PRAF2* in HaCaT, A431 and SCL-1 are detected by Western blotting. Data are presented as mean ± SD. *P < 0.05

Target genes of miR-326 were screened through bioinformatics prediction with TargetScan (http://www.targetscan.org/), Starbase (http://starbase.sysu.edu.cn/) and RegRNA (http://regrna2.mbc.nctu.edu.tw/). The intersection of the prediction results on these three datasets contained several genes. Through consulting literature, only PRAF2 is closely related to the progress of cancer [33–35]. Finally, *PRAF2* was chose for further research. Then dual-luciferase reporter gene assay was conducted to further verify the binding relationship between miR-326 and *PRAF2*. Luciferase plasmids pGL3-*PRAF2*-WT and pGL3-*PRAF2*-MUT with mutant or predicted binding sites were established, which were then used to co-transfect SCL-1 and A431 with miR-326 mimics or NC, respectively (Fig. 5a). The results unfolded that miR-326 overexpression inhibited the luciferase intensity of the WT reporter, but transfection with miR-326 caused no luciferase activity change in the MUT reporter (Fig. 5b). The above results denote that *PRAF2* is a potential target gene of miR-326. Subsequently, the expression level of *PRAF2* in CSCC cell lines was measured. As shown in Fig. 5c, in contrast to HaCaT, the control cell line, the mRNA level of *PRAF2* in CSCC cell lines was raised remarkably. Besides, the protein level of *PRAF2* was examined through Western blotting, the results of which were consistent with those of qRT-PCR (Fig. 5d).

**Fig. 6:**
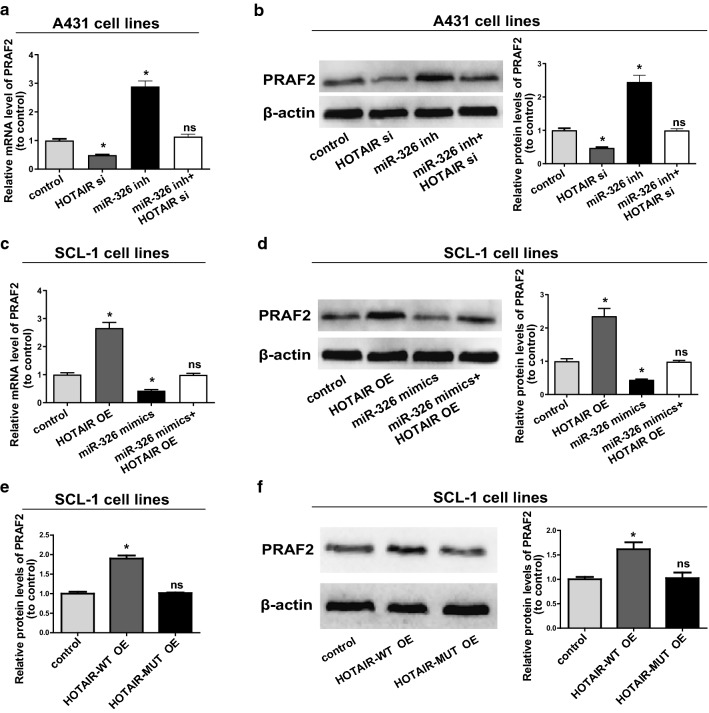
*HOTAIR*/miR-326 axis is pivotal for the expression of *PRAF2*. **a** MiR-326 inhibitors or/and *HOTAIR* siRNAs are transfected into A431 and the mRNA level of *PRAF2* is measured via qRT-PCR. **b** Western blotting analysis of *PRAF2* protein level following treatment of A431 with miR-326 inhibitors or/and*HOTAIR* siRNAs, with *β*-*actin* as a control. **c** SCL-1 is transfected with miR-326 or/and *HOTAIR* OE plasmids and the relative mRNA levels of *PRAF2* compared with controls are monitored via qRT-PCR. **d** Relative protein level of *PRAF2* in cells transfected with miR-326 mimics or/and *HOTAIR* OE plasmids. **e** Relative mRNA level of *PRAF2* in cells transfected with *HOTAIR*-WT OE plasmids or *HOTAIR*-MUT OE plasmids. **f** Relative protein level of *PRAF2* transfected with *HOTAIR*-WT OE plasmids or *HOTAIR*-MUT OE plasmids. Data are presented as mean ± SD. *P < 0.05 compared with control; ns, no significant difference

To elaborate whether *HOTAIR* was capable of modulating *PRAF2* expression level by binding to miR-326, the mRNA and protein levels of *PRAF2* in cells with regulated expressions of *HOTAIR* and miR-326 were detected. The results revealed that transfection with miR-326 inhibitors in A431 notably elevated *PRAF2* expression, whereas transfection with *HOTAIR* siRNAs exerted an opposite effect (Fig. 6a, b). Furthermore, transfection with miR-326 mimics in SCL-1 evidently repressed the expression of *PRAF2*, but transfection with *HOTAIR* OE plasmids could reverse this change (Fig. 6c, d). Subsequently, *HOTAIR* OE plasmids and its mutant overexpression plasmids were applied to transfect SCL-1, followed by determination of *PRAF2* expression. Both Western blotting and qRT-PCR results validated that overexpression of *HOTAIR*-WT upregulated *PRAF2* expression in CSCC cells, whereas *HOTAIR*-MUT did not have this effect (Fig. 6e, f). To sum up, the findings unfolded that *HOTAIR* positively modulated *PRAF2* expression by directly binding to miR-326.

### *HOTAIR*-miR-326 regulatory loop exerts a pivotal effect on cell function

**Fig. 7:**
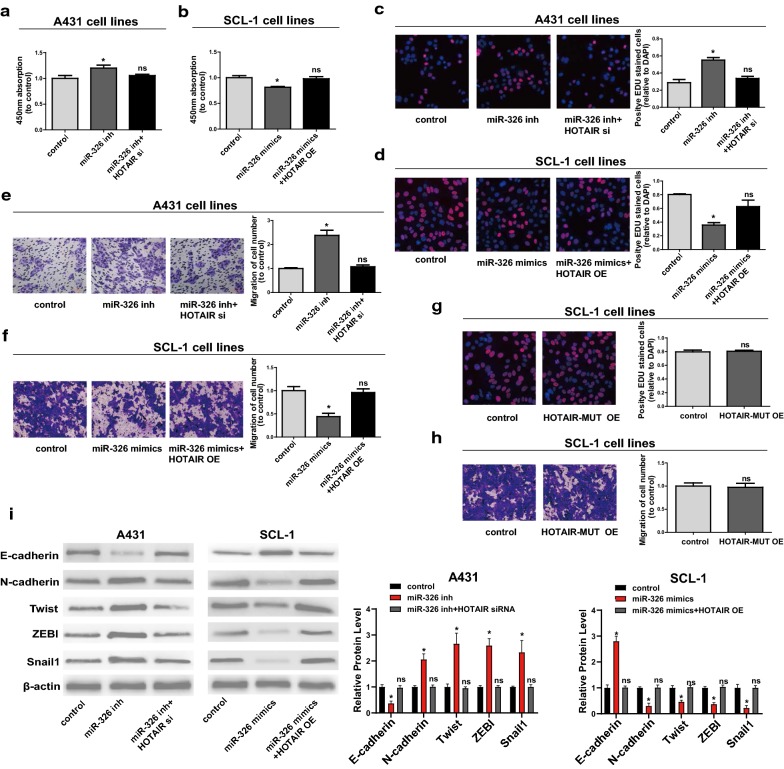
*HOTAIR* regulates cell function through miR-326. **a**, **b** CCK-8 assay is conducted to determine the proliferation of A431 and SCL-1. **c**, **d** Proliferation of A431 and SCL-1 determined via EdU assay. **e**, **f** Changes of the migration ability of A431 and SCL-1 after different transfection. **g** Proliferation of cells transfected with *HOTAIR*-MUT OE plasmids detected via EdU assay. **h** Migration of SCL-1 transfected with *HOTAIR*-MUT OE plasmids examined via Transwell assay. **i** The expression of mesenchymal marker *N*-*cadherin*, epithelial marker *E*-*cadherin* and EMT-related markers *Twist*, *Snail1 and ZEB1.* Data are presented as mean ± SD. *P < 0.05 compared with control; ns, no significant difference

**Fig. 8:**
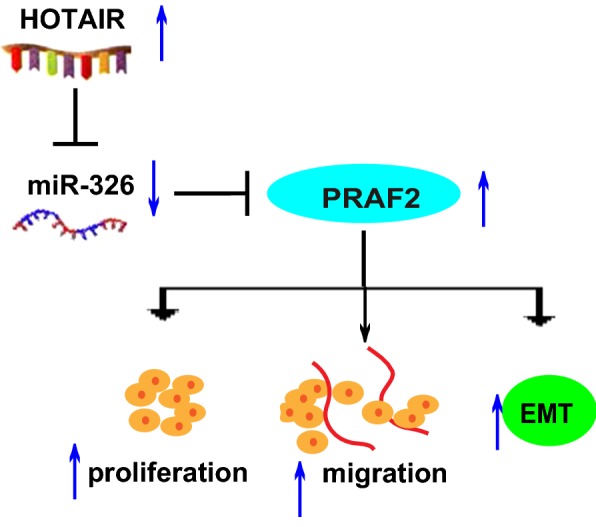
Schematic diagram of proposed mechanism. Long non-coding RNA HOTAIR may regulate cell proliferation and migration of cutaneous squamous cell carcinoma cells by acting as a competitive endogenous RNA to regulate PRAF2

Next, whether miR-326 could affect proliferative and migratory potentials of A431 and SCL-1 was explored. First of all, transfection efficiency of miR-326 mimics and inhibitors were verified by qRT-PCR (Additional file 1: Figure S1C, D). Downregulation of miR-326 in A431 markedly promoted proliferative and migratory potentials compared to controls, which were partially reversed by co-transfection with miR-326 inhibitors and *HOTAIR* siRNAs (Fig. 7a, c, e). In addition, overexpression of miR-326 inhibited proliferative and migratory potentials of SCL-1, and were partially reversed by *HOTAIR* OE (Fig. 7b, d, f). Overexpression of *HOTAIR*-MUT in SCL-1 had no effect on proliferative and migratory potentials (Fig. 7g, h). Then, we showed the expression of mesenchymal marker *N*-*cadherin*, epithelial marker *E*-*cadherin* and EMT-related markers *Twist*, *Snail1 and ZEB1* in the same experiment setting as shown in Fig. 7e, f, i). It could be seen from the above results that *HOTAIR*/miR-326/*PRAF2* axis showed great effects on regulating behaviors of CSCC cells (Fig. 8).

## Discussion

The onset of CSCC is associated with unlimited migration and proliferation of skin squamous epithelial cells [36], so any factor that affects the proliferation and migration of skin squamous epithelial cells may trigger CSCC. LncRNAs turn out to be regulators of almost all cellular processes, including cell migration and proliferation [37, 38]. Since *HOTAIR* is identified to participate in cell migration and proliferation [39, 40], it is assumed that *HOTAIR* may participate in the pathogenesis of CSCC.

The results of this study illustrated that in comparison with that in normal human keratinocyte cell line HaCaT, the level of *HOTAIR* in CSCC cell lines was raised significantly. Additionally, lowered expression of *HOTAIR* could prominently repress cell migration and proliferation as well as its EMT process, representing that *HOTAIR* is a pivotal positive regulator of CSCC cell growth and plays a role as an oncogene. Therefore, deeply investigating the mechanism of *HOTAIR* in boosting CSCC cell growth is conducive to understanding the occurrence, development and metastasis of CSCC.

Literature [31, 32, 41, 42] has demonstrated that HOTAIR is a potential ceRNA. Later, it was confirmed through RIP and dual-luciferase reporter assay that *HOTAIR* could bind to miR-326. Up to now, an enormous body of studies have shown that miR-326 expression in tumors such as colorectal cancer and gastric cancer is reduced [43, 44]. The current research proved that miR-326 was down-regulated in CSCC cell lines, and miR-326 mimics could markedly repress cell migration and proliferation. It was simultaneously found that the inhibition of miR-326 mimics on proliferation and migration of CSCC cells could be reversed by *HOTAIR* OE. All these results identify that *HOTAIR* and miR-326 may participate in CSCC occurrence and development through modulating the proliferation and migration of CSCC cells.

*PRAF2*, formerly known as JM4, is a 19-kDa protein possessing four transmembrane-spanning domains. It is a protein of the PRAF family associated with vesicle transport. In contrast to paired normal tissue samples, *PRAF2* is observed to be overexpressed in tumor tissue samples of hepatocellular carcinoma and malignant glioma [45, 46]. A study of Yco [34] has shown that *PRAF2* accelerates cell migration and proliferation and predicts poor prognosis in neuroblastoma. Correspondingly, this study manifested that up-regulated *HOTAIR* resulted in high expression of the miR-326 target *PRAF2*, which might cause abnormal migration and proliferation of CSCC cells.

## Conclusions

To sum up, this study indicates that *HOTAIR* functions as a ceRNA to modulate *PRAF2* expression by sponging miR-326, and plays a certain role in triggering CSCC.

## Supplementary information

**Additional file 1: Figure S1.** Transfection efficiency.

## Data Availability

The data in the current study are available from the corresponding authors on reasonable request.

## Abbreviations

NMSC: non-melanoma skin cancer
BCCs: basal cell carcinomas
CSCC: cutaneous squamous cell carcinoma
ceRNAs: competing endogenous RNAs
PRAF2: prenylated Rab acceptor 1 domain family, member 2
qRT-PCR: real-time quantitative PCR
IgG: immunoglobulin G
HaCaT: human keratinocyte cell line
lncRNA: long non-coding RNA
