# Supplementary material for: Long non-coding RNA HOTAIR functions as a competitive endogenous RNA to regulate PRAF2 expression by sponging miR-326 in cutaneous squamous cell carcinoma
Source: Cancer Cell Int. 2019 Oct 21;19:270. doi: 10.1186/s12935-019-0992-x (PMC6805682; doi:10.1186/s12935-019-0992-x)
Supplement: Supplementary file 1 — Additional file 1: Figure S1. Transfection efficiency. [file 12935_2019_992_MOESM1_ESM.pdf]

Figure S1

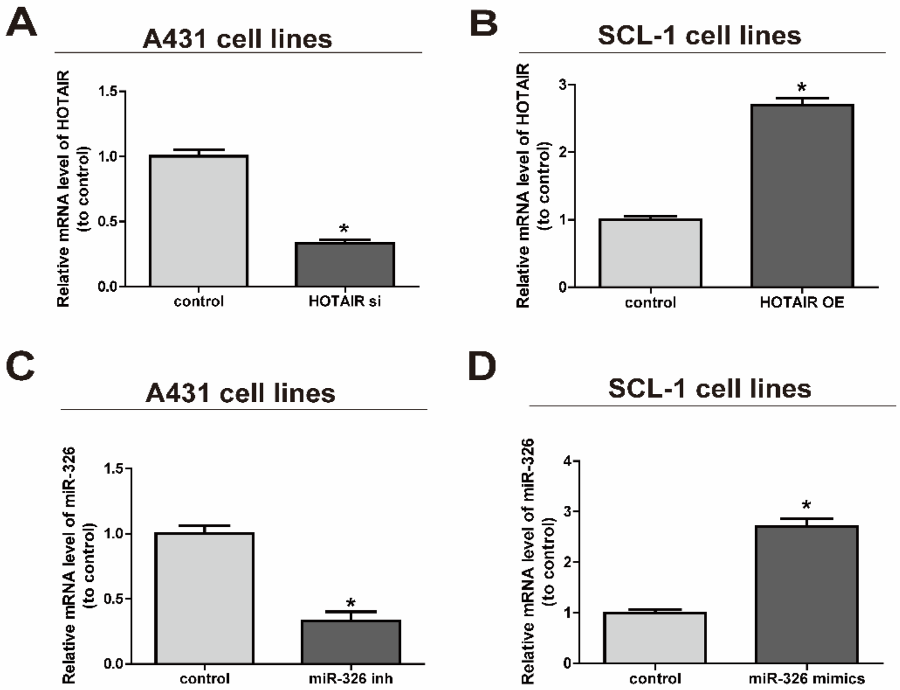

Figure S1 Transfection efficiency. (A) qRT-PCR was used to identify the effect of *HOTAIR* siRNA in A431 cells. (B) The mRNA level of *HOTAIR* in SCL-1 cells treated with *HOTAIR* over-expressing vector was detected by qRT-PCR. (C) qRT-PCR was used to identify the effect of miR-326 inhibitor in A431 cells. (D) The mRNA level of miR-326 was detected by qRT-PCR after transfection with miR-326 mimics. The data are presented as mean ± s.d. *P ⩽ 0.05, Student's t-test.
